# Pattern of secretion of pregnancy-associated plasma protein-A (PAPP-A) during pregnancies complicated by fetal aneuploidy, *in vivo* and *in vitro*

**DOI:** 10.1186/1477-7827-12-129

**Published:** 2014-12-28

**Authors:** Marie Clémence Leguy, Stephanie Brun, Guillaume Pidoux, Houria Salhi, Agnes Choiset, Marie Claude Menet, Sophie Gil, Vassilis Tsatsaris, Jean Guibourdenche

**Affiliations:** Hormonology CHU Cochin AP-HP, 27 rue du Fbg St Jacques, Paris, France; Maternity CHU Bordeaux, Place Amélie Raba-Léon, Bordeaux, France; INSERM UMR 1139, 4 av de l’observatoire, Paris, France; Foeto-pathology CHU Cochin AP-HP, 27 rue du Fbg St Jacques, Paris, France; Cytogenetic CHU Cochin AP-HP, 27 rue du Fbg St Jacques, Paris, France; PremUp foundation, 27 rue du Fbg St Jacques, Paris, France; Faculté de Pharmacie, Université Paris Descartes, 4 av de l’observatoire, Paris, France; Maternity CHU Cochin AP-HP, 27 rue du Fbg St Jacques, Paris, France

**Keywords:** Pregnancy-associated placental protein-A, Villous cytotrophoblast, Maternal serum, Fetal trisomy, Ultrasound scanning, Placental dysfunction

## Abstract

**Background:**

Pregnancy-associated placental protein-A (PAPP-A) is a metalloprotease which circulates as an hetero-tetramer in maternal blood. Its maternal serum concentration in fetal trisomy 21 is decreased during the first trimester, so that PAPP-A is a useful screening biomarker. However, the regulation of PAPP-A placental secretion is unclear. We therefore investigated the secretion of PAPP-A in pregnancies complicated by fetal aneuploidies, both *in vivo* and *in vitro*.

**Methods:**

Maternal serum collected between 10 WG and 33 WG during 7014 normal pregnancies and 96 pregnancies complicated by fetal trisomy 21, 18, and 13 were assayed for PAPP-A using the Immulite 2000xpi system®. The pregnancies were monitored using ultrasound scanning, fetal karyotyping and placental analysis. Villous cytotrophoblasts were isolated from normal and trisomic placenta and cultured to investigate PAPP-A secretion *in vitro* (n = 6).

**Results:**

An increased nuchal translucency during the first trimester is a common feature of many chromosomal defect but each aneuploidy has its own syndromic pattern of abnormalities detectable at the prenatal ultrasound scanning and confirmed at the fetal examination thereafter. PAPP-A levels rise throughout normal pregnancy whereas in trisomy 21, PAPP-A levels were significantly decreased, but only during the first trimester. PAPP-A levels were decreased in trisomy 13 and sharply in trisomy 18, whatever the gestational age. *In vitro*, PAPP-A secretion was decreased in aneuploidy, and associated with decreased hCG secretion in Trisomy 21 and 18. These biochemical profiles did not appear to be linked to any specific histological lesions affecting the placenta.

**Conclusions:**

These profiles may reflect different quantitative and qualitative placental dysfunctions in the context of these aneuploidies.

## Background

Pregnancy-associated placental protein-A (PAPP-A) was first isolated in the 1970s from the serum of pregnant women, and the synthesis of PAPP-A mRNA has since been demonstrated in numerous tissues [1, 2]. PAPP-A is a metalloprotease belonging to the metzincin superfamily of zinc peptidase. It is active as a homodimer (dPAPP-A) which cleaves insulin-like growth factor binding proteins 4 and 5, thus regulating local IGF bioavailability and hence cell differentiation and proliferation [3, 4]. dPAPP-A circulates at very low levels in non-pregnant women and men [5]. This dPAPP-A is abundantly expressed in unstable coronary atherosclerotic plaques and can be released in the event of rupture, thus rendering dPAPP-A a biomarker for coronary syndrome and unstable angina [6, 7]. In pregnancy, PAPP-A is produced at high levels by the placenta and circulates as a covalent 2:2 heterotetrameric complex (htPAPP-A), designated PAPP-A/proMBP, with the proform of an eosinophil major basic protein (proMBP) that inhibits dPAPP-A proteasic activity [8]. Measurement of this complex is of interest in pregnancy [9, 10]. Decreased levels are associated with adverse pregnancy outcomes such as intrauterine growth restriction (IUGR), preterm delivery, miscarriage and pre-eclampsia [10–12]. The principal routine value of PAPP-A is the prenatal screening of fetal aneuploidy, particularly for trisomy 21 which is often associated with low PAPP-A levels during the first trimester of pregnancy [13, 14]. However, the patho-physiological mechanisms underlying this decrease are unclear and little is known about PAPP-A levels during the second and third trimesters of pregnancy in the event of fetal aneuploidy. Our aim was therefore to investigate the placental secretion of PAPP-A *in vivo* by characterising maternal levels of PAPP-A throughout pregnancy in the case of fetal aneuploidy (trisomy 21, trisomy 18 and trisomy 13) in a large cohort, and *in vitro* by culturing villous cytotrophoblasts isolated from aneuploid placentas.

## Methods

### Maternal serum collection

Maternal blood samples were collected prospectively according to the French policy on the screening of prenatal trisomy 21 at Port Royal Maternity Department Hospital, which is a reference centre for prenatal screening in Paris. This type of screening must be offered to every pregnant woman since 10 WG (weeks of gestation) [15]. Calculating the risk requires the measurement of nuchal translucency thickness (nt) and maternal serum markers i.e. pregnancy-associated plasma protein-A (PAPP-A) and the free β subunit of chorionic gonadotrophin (hCGβ) before 14WG; after that time point, hCGβ or total hCG and alphafoetoprotein (AFP) are determined, in combination with maternal age. The risk of T21was calculated using the Immulite 2000 automatic system and Prisca 4 software (Siemens® Typolog, Germany) [16]. Additional maternal blood samples collected at the time of pregnancy terminations in the cases of isolated major fetal abnormalities were included in the study (i.e.: cardiac or, renal or cerebral abnormalities of very poor prognosis). All blood samples were centrifuged for 10 min. at 4000 G at room temperature and then frozen until assay. We also reviewed fetal ultrasound profiles during the first trimester and after if available, fetal karyotyping when it was performed, and the immediate neonatal outcome. The overall population was divided into two groups: control pregnancies (n = 7014, pregnancies not associated with any maternal complication at the time of sampling and fetal aneuploidy) and pregnancies complicated by fetal aneuploidy (n = 96: trisomy 21 n = 68, trisomy 18 n = 21, and trisomy 13 n = 7) from 10WG to 33WG. Among those cases of aneuploidy, 55 cases were fully documented (trisomy 21 n = 31, trisomy 18 n = 21, and trisomy 13 n = 3).

### Foeto-placental examination and tissue collection

French law allows for the termination of pregnancy with no gestational age limit when severe fetal abnormalities are observed. Placenta specimens were collected at the time of the termination of pregnancy and underwent macroscopic and microscopic examination. Pathologists analysed aneuploid placentas in 55 aneuploid cases at both the macroscopic level (i.e.: hypotrophy, immaturity, sponginess, hydropsy/ oedema, single umbilical artery), and histological level (principally hydropic villi, immature villi, post-mature villi, calcifications, fibrin deposits, trophoblastic cysts, bullous dystrophy) after being fixed in formalin, included in paraffin and stained with hematein-eosin-saffron. Each item was graded (0 if absent, 1 if present, 2 if abundant) in order to establish a histological score. We were able to isolate villous cytotrophoblasts from fresh placentas for *in vitro* culture in three cases: one of trisomy 21 at 20 WG, one of trisomy 18 at 19WG and one of trisomy 13 at 18WG, and three gestational age-matched controls. Gestational age was confirmed by ultrasound measurement of the crown-rump length at 8–12 WG. Trisomy 21, 18 and 13 were diagnosed by karyotyping chorionic villi, amniotic fluid cells or fetal blood cells. In no case was aneuploidy due to translocation, and no mosaicism was observed.

### Cell cultures

Cytotrophoblast cells were isolated after trypsin-Dnase I digestion and discontinuous Percoll gradient fractionation, as described by Kliman but with slight modifications [17, 18]. Briefly, the villous sample was subjected to sequential enzymatic digestion in a solution containing 0.5% trypsin powder (W/V, Difco), 5 IU/ml DNAseI, 25 mM HEPES, 4.2 mM MgSO_4_ and 1% (W/V) penicillin/streptomycin (Biochemical industrie) in HBSS, with monitoring by light microscopy. The first digest, and in some cases the second digest, were discarded on the basis of the light microscopy findings, in order to eliminate any syncytiotrophoblast fragments. The cells contained in the four or five subsequent digests were purified on a discontinuous Percoll gradient (5% to 70% in 5% steps). Cells that sedimented in the middle layer (density 1.048-1.062 g/ml) were further purified using a monoclonal anti-human leukocytic antigen A, B and C antibody (W6-32HL, Sera Lab, Crawley Down, UK). W6-32HL negative cytotrophoblast cells were diluted to a final density of 0.5x10^6^/ml in Dulbecco's modified Eagle's medium (DMEM) containing 10% foetal calf serum (FCS). After 4 hours of incubation at 37°C under 5% CO_2_, non-adherent cells and syncytial fragments were removed by three efficient washes in culture medium. After 3 hours of culture, 95% of cells isolated from full-term placentas were cytotrophoblastic cells, as shown by positive cytokeratin 7 staining with a specific monoclonal antibody (dilution 1:200, Dako). The cells were further cultured in 2 ml of DMEM supplemented with 25 mM HEPES, 2 mM glutamine, 10% heat-inactivated FCS and antibiotics (100 IU/ml penicillin and 100 mg/ml streptomycin) at 37°C under humidified 5% CO_2_-95% air. The culture supernatants of aneuploid villous cytotrophoblasts were then integrated in the placental physiopole collections operated by the Perinatcollection project (ANR).

### PAPP-A assay

PAPP-A concentrations in the maternal serum and culture medium were determined using the Immulite 2000 xpi automated chemiluminescent analyser® (Siemens, Germany) [16]. The IMMULITE 2000 PAPP-A assay is a solid–phase enzyme-labelled chemiluminescent immunometric assay that uses two monoclonal mouse anti-PAPP-A antibodies. This assay was first standardized against the Brahms Kryptor PAPP-A assay. Its analytical sensitivity was 0.025 mU/ml; the within- and between-run coefficients of variation were below 4% and 12%, respectively, and the calibration range was 0–10 mU/ml. Total hCG was also measured in culture medium as an index for formation of the endocrine syncytiotrophoblast using an Advia Centaur XP analyser® (Siemens, Germany).

### Data analyses

Statistical analysis were performed using the Statview F-4.5 software package® (Abacus Concepts, Inc., Berkeley, CA, USA). Values are expressed as crude values and 10ciles, 50ciles, 90ciles values. Significant differences (p < 0.05) were identified using the non parametric Kruskal-Wallis and Mann–Whitney U tests.

## Results

### Ultrasound scanning characteristics of fetal aneuploidies

In the 55 cases fully documented, ultrasound scanning is informative from the first trimester, particularly in trisomy 18 (17/21; 81%) (see Additional file 1, Table 1). Around one third of the trisomic 18 fetuses presented at least two associated abnormal signs at ultrasound scanning. The most frequent signs were enlarged nt, exomphalos and cystic hygroma. Other signs tend to become detectable during the second and third trimesters of pregnancy, mainly in the case of Trisomy 21 even if there were not particularly associated with any type of aneuploidy.

**Table 1:** **Common sonographic defects in aneuploides fetuses**

	Trisomy 21 (n = 31)	Trisomy 18 (n = 21)	Trisomy 13 (n = 3)
1rst trimester			
Cystic hygromata	2	3	0
Enlarged NT (≥3.5 mm)	14	9	0
Hydrops	0	1	0
Cardiac abnormalities	0	2	1
Encephalocele	0	2	0
Spina bifida	0	2	0
Exomphalos	0	5	1
2d and 3d trimester			
Choroid plexus cysts	1	2	0
Other brain abnormalities	1	0	1
Skull and face abnormalities	1	1	0
Cardiac abnormalities	3	2	1
Digestive atresia	1	1	0
Hepatic calcifications	1	0	0
Renal abnormalities	0	1	0
Ascite	0	1	0
Extremities abormalities	1	1	1
Short femur	1	1	0
Macrosomia or hypotrophia	1	1	0
Fetal death	0	1	0
Decrease or increased amniotic fluid volume	4	2	1

Ultrasound scanning was performed by experimented operators at the first, the second and the third trimester on 55 fetuses which were thereafter diagnosed trisomic 21, trisomic 18 and trisomic 13 diagnosed by karyotyping chorionc villi, amniotic fluid cells or fetal blood cells.

### Maternal serum PAPP-A levels during the course of pregnancy

**Figure 1:**
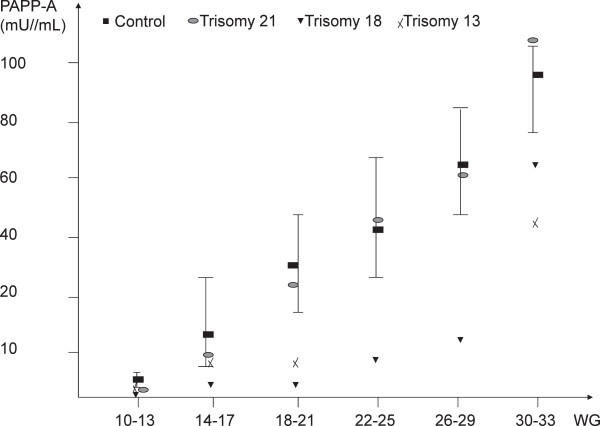
**Maternal serum levels of PAPP-A throughout normal gestation and in gestations complicated by fetal aneuploidy.** PAPP-A was measured using the assay developed on the Immulite 2000 analyser (Siemens, Germany) specific for the heterotetrameric complex (htPAPP-A) in the maternal serum of 7014 normal pregnancies (controls) and 96 pregnancies affected by fetal trisomy (68 trisomy 21, 21 trisomy 18, 7 trisomy 13) from 10WG to 33WG. Results are expressed in median values, and 10-90ciles for the controls.

PAPP-A levels rise throughout a normal gestation from 2.3 mU/mL at 10WG to 56 mU/ml at 24WG and reaches 103 mU/mL at 33WG (Figure 1). There is a sharp, 24-fold increase (p = 0.01) between the end of the first trimester and the end of the second trimester, followed by a slower, 2-fold increase from the end of the second trimester until delivery. In trisomy 21 PAPP-A levels compared with the controls were significantly decreased at the end of the first trimester (median value: 2.7 mU/mL, p = 0.02 ) but not thereafter (45.8 mU/mL at the end of the second trimester and 120 mU/mL near the term). In trisomy 18 and trisomy 13, maternal PAPP-A levels were always significantly lower (p < 0.01 and p = 0.02 respectively) than in the controls whatever the gestational age. PAPP-A levels were particularly low in trisomy 18 (median value: 0.9 mU/mL at the end of the first trimester, 9.7 mU/mL at the end of the second trimester and 67.7 mU/mL near the term)

### *In Vitro*secretion of PAPP-A and hCG by villous trophoblastic tissue

**Figure 2:**
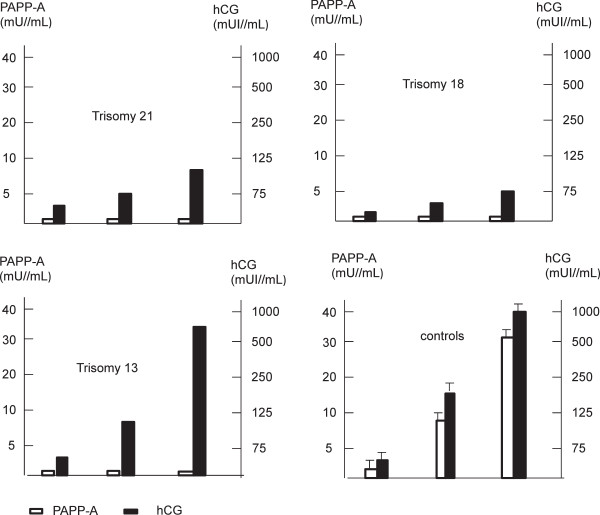
***In vitro*** **secretion of PAPP-A and hCG by villous trophoblast tissues isolated from normal placentas and placentas complicated by aneuploidy.** Villous cytototrophoblasts were cultured after being isolated from aneuploid placentas (one trisomy 21 at 20 WG, one trisomy 18 at 19WG, one trisomy 13 at 18WG) and from three age matched normal placentas. PAPP-A was measured in the culture supernatant from 24 hours to 72 hours of culture using the assay developed on the Immulite 2000 analyser® (Siemens, Germany) specific for the heterotetrameric complex (htPAPP-A). hCG was measured with the assay developed on Advia Centaur XP analyser® (Siemens, Germany) to assess the formation of the syncytiotrophoblast.

We measured the PAPP-A and total hCG secreted *in vitro* by villous cytotrophoblasts during their differentiation into syncytiotrophoblast. In the controls, PAPP-A and hCG levels increased concomitantly with the formation of syncytiotrophoblast from 24 to 72 hours of culture, by 10 and 20-fold respectively. hCG secretion was significantly decreased in trisomy 18 and to a lesser extent in trisomy 21 but not in trisomy 13. The secretion of PAPP-A was significantly very low (<1 mU/mL, p = 0.01) in the context of any aneuploidy, with no increase during the culture (Figure 2).

### Placental histology

**Figure 3:**
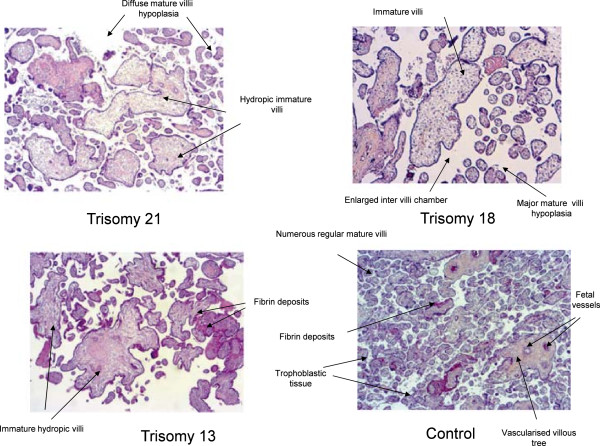
**Microscopic analysis of placental villi in normal pregnancy and pregnancy complicated by aneuploidy.** After termination of pregnancy, 55 aneuploid placentae were analysed by experimented pathologists at the macroscopic level, and the histological level after being fixed in formalin, included in paraffin and stained with hematein-eosin-saffron. Mature villi i.e. small diameter, strong staining, presence of fetal vessels, decreased connective tissue, covered by trophoblastic tissue. Immature hydropic villi i.e. large diameter, pale staining, few fetal vessels, connective tissue, decreased trophoblastic tissue.

Investigations were performed in 55 patients (see Additional file 2, Table 2). Although it was inconsistent, the placenta was macroscopycally rather small (28/55, 51%) and abnormal (44/55, 80%). In trisomy 21 and trisomy 18, it presented an abnormal mesenchymal core and abnormal trophoblastic tissue when compared to age matched controls (Figure 3). Indeed, the placental villi displayed an important immaturity (26/55, 47%) with an increased mean villous diameter and stromal hydrops; a reduction in the number of vessels; a trophoblastic tissue irregular associating a hypoplasic syncytium with a persisting double layer of villous cytotrophoblasts. These cytotrophoblasts were lying on frequent fibrin deposits (16/55, 29%), calcifications (10/55, 18%), hydropic areas (49/55, 89%) and basophil infiltrates. Hydropic villi and calcifications were also observed in trisomy 13. A single umbilical artery and of trophoblastic cysts were only noted in the context of trisomy 18 whereas bullous dystrophy was only noted in the context of trisomy 21. No abnormality was specific of trisomy 13. The median histological scores were 6 for Trisomy 21 and 5 for Trisomy 18 (data not shown).

**Table 2:** **Principal macroscopic and microscopic findings regarding placentas complicated by aneuploidy**

	Trisomy 21 (n = 31)	Trisomy 18 (n = 21)	Trisomy 13 (n = 3)
Macroscopy			
Hypotrophy	16	12	0
Immaturity	24	14	0
Sponginess	20	10	0
Hydropsy	24	20	0
Single umbilical artery	0	4	0
Histology			
Hydropic villi	28	19	2
Immature villi	20	6	0
Post-mature villi	12	6	0
Calcifications	4	4	2
Fibrin deposits	12	4	0
Trophoblastic cysts	0	4	0
Bullous dystrophy	8	0	0

## Discussion

This work confirms the value of combining an early determination of maternal serum markers such as PAPP-A with prenatal ultrasound scanning in order to detect fetal aneuploidy [19–21]. Indeed, phenotypic abnormalities exist in both trisomic fetuses and the placenta [22, 23]. The anatomical differences between the controls and trisomic patients are significant enough to be detected *in vivo* using prenatal ultrasound scans. Post-mortem examinations of affected fetuses have confirmed such differences [24].

As expected, during the first trimester, a common feature of many chromosomal defects is increased nuchal translucency [25]. However, our work has shown that each chromosomal defect tended to have its own syndromic pattern of abnormalities that affect the skull and brain, the face and neck, the chest, abdomen and extremities [26].

Although ultrasound scans can demonstrate that major chromosomal defects are often associated with multiple fetal abnormalities, maternal serum markers may provide valuable additional information [26]. We confirm here in a large cohort that fetal aneuploidies, are generally associated with decreased PAPP-A levels in maternal serum [27–29]. This reduction was limited to the first trimester of pregnancy in trisomy 21 where as it became significative in the second half of pregnancy in case of trisomy 13. We were able to establish that it persists throught the gestation in trisomy 18. These biochemical profiles did not appear to be linked to any specific histological lesions affecting the placenta. The present study has thus confirmed that in a context of aneuploidy, the villi are mainly immature, hydropic and poorly vascularised, with fibrin deposits but with large variability [30–34]. The trophoblastic tissue is poorly developed with a thin syncytiotrophoblast and the persistence of a double layer of villous cytotrophoblasts. The patterns of trisomic placental villi is known to change between the first and second trimesters of pregnancy. During the second trimester, trisomic villi were predominantly large, irregular and hypovascular, while during the third trimester, this type of villus abnormality was only observed in a few villi and was associated with focal hypervascularity.

It is likely that the decrease in PAPP-A levels is not directly related to the chromosomal defect because the PAPP-A gene is located on human chromosome 9 and not on chromosomes 21, 18 or 13. The protein is secreted as an active dPAPP-A homodimer in the form of a metalloproteinase which both interacts with the extracellular matrix and cleaves IGFBP-4 and 5, thus increasing the local bioavailability of IGFs [2–4, 8, 35–38]. It has different features that allows PAPP-A to interact with laminin, complement and heparin sulphates on the cell surface and in the extra-cellular matrix. During pregnancy, almost all circulating PAPP-A is bound covalently to a glycoprotein, proMBP (preform of eosinophil major basic protein) to form a hetero-tetrameric complex composed of two PAPP-A and two proMBP subunits [39]. We were able to establish the maternal profile of htPAPP-A first using antibodies specific to this complex, and second not only during the first trimester but also during the second and third trimesters. We confirmed the sharp increase of PAPP-A during the first half of pregnancy, which has been suggested to reflect the increase in placental volume, and we showed that the slope of this increase rose very slowly during the second half of pregnancy [40–42]. However, very little is known as yet about the individual secretion profiles of dPAPP-A and proMBP [43]. The latter is believed to occupy the cell-surface binding site of PAPP-A in the circulating complex, so that the tetramer can't bind to the cell surface when it enters the maternal circulation. dPAPP-A and proMBP, and the htPAPPA heterotetrameric complex are expressed physiologically in the villous trophoblast of the first and third trimesters [4, 44–47]. dPAPP-A is weakly expressed in the ST at full-term, whereas htPAPP-A displays the opposite pattern. We had previously explored the pattern of PAPP-A secretion by the villous trophoblast *in vitro*, showing that this secretion increased in line with formation of the endocrine syncytiotrophoblast [48]. During the present study, we investigated the pattern of PAPP-A secretion by the aneuploid villous trophoblast. Our preliminary results suggest that in the case of aneuploidy, the villous trophoblast secretion of PAPP-A was altered. In the case of trisomy 18, we observed an *in vitro* decrease in hCG and PAPP-A secretion, and *in vivo* a small placental mass and syncytial mass. We also established *in vivo* that in trisomy 18, decreased maternal serum PAPP-A levels were not restricted to the first part of pregnancy. Thus, low maternal PAPP-A levels levels are likely to reflect both a reduction in placental volume and lower levels of trophoblastic secretion [49–51]. In trisomy 13, as the placental mass is normal, we can speculate that the decreased maternal serum PAPP-A levels we observed all along the pregnancy result predominantly from a defective trophoblastic production. In trisomy 21, we confirmed our previous *in vitro* findings regarding defective differenciation of villous cytotrophoblasts into a syncytiotrophoblast [52]. This led to the decreased PAPPA secretion we observed. However, we confirmed that *in vivo*, maternal serum PAPP-A levels were decreased but only in the first trimester of pregnancy. Thus, the mechanisms involved may be more complex and may differ from one aneuploidy to another [53].

This PAPP-A decrease seems to be related to a global an impairment of satisfactory differentiation of the villous cytotrophoblast but also of the extravillous cytotrophoblast Indeed, because the decrease in PAPP-A levels is observed early in any pregnancy associated to fetal aneuploidy, the defect may concern the extravillous cytotrophoblast, which is the principal source of PAPP-A at this stage. We had previously demonstrated *in vitro* changes to the patterns of PAPP-A secretion, to their regulation during normal gestation and to the trophoblastic phenotype, i.e. villous cytotrophoblast or extravillous cytotrophoblast [54]. Further studies are now necessary to focus on aneuploid extravillous cytotrophoblasts, but some findings have suggested defective differentiation along the invasive pathway that can affect not only PAPP-A but also other proteases (i.e. MM-9, ADAM12) [55, 56]. This could explain why these proteases have been proposed as biomarkers of aneuploidy [57]. It is likely that not only the trophoblastic cells but all cells in the villi could turned over and be differentially modified in the context of aneuploidy [30, 58, 59].

## Abbreviations

AFP: Alphafoetoprotein
hCG: Chorionic gonadotrophin
PAPP-A: Pregnancy-associated placental protein-A
WG: Weeks of gestation.
